# Trends in HIV prevalence and risk factors among men who have sex with men in Mozambique: implications for targeted interventions and public health strategies

**DOI:** 10.1186/s12889-024-18661-0

**Published:** 2024-04-27

**Authors:** Áuria Ribeiro Banze, Rachid Muleia, Samuel Nuvunga, Makini Boothe, Cynthia Semá Baltazar

**Affiliations:** 1 National Institute of Health, EN1, Bairro da Vila- Parcela nº 3943 Marracuene District, Maputo, Maputo Province Mozambique; 2 UNAIDS, Maputo, Mozambique

**Keywords:** HIV, Prevalence, MSM, Trends, Mozambique

## Abstract

**Background:**

Men who have sex with Men (MSM) are known to contribute to increased HIV prevalence as an integral part of key populations with high vulnerability to HIV/AIDS due to their sexual behaviours. Mozambique conducted two rounds of bio-behavioral surveys (BBS) in this population with the main objective of estimating HIV prevalence and associated risk behaviors among MSM in Mozambique. The present study aims to estimate the trend of HIV prevalence and determine the correlations of HIV infection among MSM.

**Methods:**

A repeated cross-sectional analytical study was conducted from secondary data from the first and second rounds of BBS in Mozambique conducted in 2011 and 2020–2021 respectively. Each round used a similar methodology which allowed for comparison between the two surveys. Chi-square test and logistic regression was used to compare the HIV prevalence between the BBS rounds, identify factors associated with HIV, and assess changes in HIV prevalence across selected risk factors.

**Results:**

There was a significant increase in HIV prevalence among MSM (7.1–14.9%), living in Maputo (9.3–14.7%), uncircumcised (11.7–25.1%), and those who reported two sexual partners in the last year (5.2–14.4%). In contrast, there was a decrease in prevalence among adult MSM aged between 25 and 29 years (24.7–13.9%), aged 30 years or older (45.7–27.7%), married (29.1–16.8%), with higher education (16.7–5.9%) and moderate perception of HIV risk (10.9–3.4%). Multivariable analysis showed that factors such as age, marital status, religion, sexual identity, circumcision, and the use of lubrication during anal sex are significantly associated with the risk of HIV infection.

**Conclusions:**

This study underscores the continuing need for HIV prevention and education efforts. The rise in prevalence among specific population segments and the sustained presence of risk factors emphasize the requirement for holistic strategies tailored to the unique requirements of each subgroup. Understanding trends and risk factors is crucial to guiding public health policies and designing efficacious prevention programs that aim to curtail HIV transmission while enhancing the well-being of those impacted by the condition.

## Introduction

Despite significant advancements in HIV treatment coverage and the expansion of access to HIV prevention initiatives, the global HIV epidemic persists as a critical public health challenge, characterized by concerning incidence rates in numerous countries. At present, an estimated 38 million individuals worldwide are living with HIV, and Sub-Saharan Africa stands as the epicenter of the HIV/AIDS outbreak, accounting for over two-thirds of the global burden of new HIV infections [1, 2].

The key populations and their sexual partners are recognized as significant drivers of HIV infections, collectively accounting for 70% of the global tally of new HIV infections [2]. Within these populations, gay men and other men who have sex with men (MSM) are particularly affected, being 28 times more likely to contract HIV than the general population [1]. This statistic, however, should not be viewed solely as an epidemiological data point. Rather, it underscores the critical need to address health and wellbeing concerns specific to the MSM community.

Recent studies reveal the prevalent presence of MSM throughout the sub-Saharan Africa region, accompanied by elevated rates of HIV infection [3, 4]. The heightened susceptibility of this population to HIV is due to high levels of stigma, criminalization, and homophobia, that this group experiences due to their sexual orientation and practices, which hampers public health interventions as well as the availability and accessibility of HIV prevention programs [3, 4].

Mozambique stands among the countries with a substantial HIV burden, with a generalized HIV epidemic and an estimated prevalence of 12.5% among individuals aged 15–49 years old, as indicated by the most recent Population-based HIV Impact Assessment (PHIA) survey conducted in 2021 [5]. The Mozambique National HIV Strategic Plan (PEN) recognizes MSM as a priority group for targeted HIV/AIDS interventions [6]. Mozambique carried out two rounds of Bio-behavioral Surveys among MSM using Responded Driven Sampling (RDS) methodology. The first was conducted in 2011 and found a prevalence 8.2% (CI: 4.7–12.6%) in Maputo, 9.1% (CI: 5.8–12.6%) Beira, and 3.1% (CI: 1.1–7.1%), Nampula/Nacala cities. Among MSM, 8.2% in Maputo City, 9.3% of in Beira and 3.7% in Nampula were aware of their HIV status [7]. During 2020–2021, the country implemented the second BBS among MSM in 5 main urban areas.

Despite significant progress to contain the HIV epidemic over the past decade, Mozambique is unlikely to achieve the objectives of the 95-95-95 Global Fast-Track Strategy, aiming to end HIV by 2030. Furthermore, the coronavirus disease 2019 (COVID-19) pandemic has exacerbated the challenges faced by many nations in sustaining progress in HIV control [2]. In light of these circumstances, this study aims to evaluate the trajectory of HIV prevalence and associated risk behaviors among MSM in Mozambique, contributing to informed decision-making rooted in evidence for targeted HIV prevention within this population.

## Methods

### Study design

The analysis in this study was based on data from the two cross-section surveys among MSM conducted in Mozambique: the first round in 2011 [7, 8] and the second in 2020 [9]. The first BBS was carried out in three primary urban areas—Maputo, Beira, and Nampula—while the subsequent 2020 BBS was extended to include two additional cities, Tete and Quelimane. These surveys were designed to collect a wide range of information on socio-demographic characteristics, behavioural factors related to HIV and other sexually transmitted diseases (ex: sexual history, condom use, alcohol, and other drug use, etc.), health services access and healthcare seeking behaviour.

In the context of this manuscript, our analysis has been restricted to the data extracted from the two survey rounds conducted exclusively in Maputo, Beira, and Nampula to allow comparability between surveys.

### Study population and sample size

In each of the survey, MSM were eligible to participate on the survey if they were biological male, aged ≥ 18, reported having engaged in either oral or anal intercourse with another man in the last 12 months before the survey, and lived, worked, or socialized in the last six and 12 months for the 2011 BBS and 2020 BBS, respectively.

The first round of BBS recruited 496 from Maputo, 496 from Beira and 583 from Nampula/Nacala. In the second round, 530 MSM were recruited in Maputo, 527 in Beira, and 528 in Nampula. For the purposes of this analysis, we considered data from MSM with a valid HIV test result. As a result, data analysis includes 1379 MSM from the 2011 BBS, and 1580 MSM from the 2020 BBS. In both surveys, study participants were recruited using RDS, a methodology designed to effectively sample populations that are traditionally challenging to access. More details about the survey methodology are described elsewhere [10].

### Data collection and measures

Socio behavioral data were collected using a standardized questionnaire that includes socio-demographic characteristics, risk behavioral related to HIV and other sexually transmitted infections (STIs), sexual history, number of sexual partners, condom use, and alcohol and substance abuse. Additionally, the questionary includes knowledge and healthcare seeking behavior.

The sexual identity was classified into categories - bisexual, gay/homosexual, heterosexual, transexual. The practices of anal sex were classified as either “insertive penile sex” or “receptive penile sex” within the preceding 12 months. Participants self-reported STI was assessed by the 3 following questions: “During the last six months, have you had an abnormal discharge from your anus or penis?”, “During the last six months, have you had a sore or ulcer near your anus or penis?,” and “In the last six months, did someone inform you that you had or could have a sexually transmitted infection?

Biological data were collected for on-site rapid test for HIV, syphilis, and Hepatitis B virus (HBV). The HIV rapid test procedures followed the national sequential testing algorithm using Alere Determine® HIV-1/2 (Abbott Laboratories, UK), followed by a confirmatory test using Uni-Gold™ HIV (Trinity Biotech, Irland). Syphilis and HBV was diagnosed using a rapid test (SD Bioline 3.0®, Standard Diagnostics and Alere Determine® HbsAg, Abbott Laboratories, respectively).

### Data management and analysis

Data were analyzed using R statistical software (version 4.2.1). Point estimates and confidence intervals for the HIV prevalence were computed using the RDS R package, a module which is part of the RDS Analyst (RDS-A) suite of packages for the analysis of respondent-driven sampling data (REF). The RDS R package offers several estimators for producing point estimates. Nevertheless, for this study we use the Giles successive sampling (RDS-SS) estimator. For comparison of prevalences of HIV between the two surveys, we used a Chi-square test for equality of proportion.

Descriptive statistics were conducted for demographic, behavioral, and biological variables. To measure associations between HIV sero-status and predictor variables, bivariate logistic regression analysis was performed, with a p-value ≤ 0.05 designated as the cutoff for inclusion in multivariate logistic regression models. The multivariate logistic regression model was used to evaluate changes in HIV infection among MSM over nine years, where the HIV sero-status of participants was taken as the outcome variable.

Moreover, to understand how changes in the risk of HIV infection varied by each independent variable, we tested for interactions. We considered all interactions between the variable ‘year’ and each independent variable. Additionally, we used a stepwise variable selection method to remove less important variables and interactions in the multivariate logistic regression. In this analysis, we considered unweighted logistic regression, as many studies suggest that this approach outperforms logistic regression with RDS [11, 12]. Statistical significance was set at a 5% significance level.

### Ethical considerations

Written informed consent for all eligible participants was required. Both BBS protocols were approved by the Institutional Bioethics Committee (CIBS) and the National Bioethics Committee for Health (CNBS). This analysis was approved by the National Institute of Health – Data Management Unit and the analyses were performed in accordance with ethical principles and regulatory bodies and in accordance with internationally established standards where applicable.

The consent process was designed to allow participants to give separate consent for certain components of the survey. This included the option to not undergo rapid HIV diagnosis but only participate in answering the questionnaire.

## Results

### Trends in HIV prevalence by demographic characteristics and risk factors

Table 1 presents the descriptive analysis showing the changes in HIV prevalence by selected characteristic for BBS implemented in 2011 and 2020. From 2011 to 2020, HIV prevalence has significantly increased among MSM gay/homosexual (7.1–14.9%, 95% CI: -11.46;-4.1, *p* < 0.001), from Maputo (9.3–14.7%, 95% CI: -9.5;-0.98, *p* = 0.017), non-circumcised (11.7% to 25.1, 95% CI: -21.64; -5.20, %*p* < 0.001), those who reported 2 sexual partners in the last year (5.2–14.4%, 95% CI: -9.31;-0.97, *p* < 0.001).

The results suggest a decline in HIV prevalence among adult MSM 25–29 (24.7–13.9%, 95% CI: 2.86; 18.14, *p* = 0.004) years old and 30 years old or more (45.7–27.7%, 95% CI: 4.32; 30.82, *p* = 0.007), married (29.1–16.8%, 95% CI: 0.61;23.96, *p* = 0.034), with higher education (16.7–5.9%, 95% CI: 2.37; 19.9, *p* = 0.016), and those with a moderate perception of HIV risk (10.9 to 3.4%, 95% CI: 2.91; 12.28, *p* = 0.001).

**Table 1:** Distribution of HIV prevalence by selected variables for 2011 and 2020 BBS

Characteristics	2011 BBS		2020 BBS		Difference of prevalences	
	*n/N*	% (95% CI)	*n/N*	% (95% CI)	95%CI	*p*-value
**Age**						
18–19	7/454	1 (0.2;1.7)	17/371	3.5 (1.4;5.7)	(-4.75;-0.05)	0.035
20–24	29/636	4.1 (1.7;6.4)	69/753	6.4 (3.6;9.1)	(-4.76;0.19)	0.077
25–29	38/203	24.7 (17.0;32.3)	48/276	13.9 (8.2;19.7)	(2.86;18.14)	0.004
30+	40/86	45.7 (33.3;58.0)	44/180	27.7 (18.9;36.4)	(4.32;30.82)	0.007
**Education**						
Primary	24/198	12.4 (6.4;18.5)	1/8	7 (0;22.3)	(-23.38;23.63)	1
Secondary	67/1047	6.2 (3.1;9.3)	45/726	4.7 (2.9;6.5)	(-0.71;3.76)	0.204
Higher education	22/125	16.7 (7.7;25.7)	10/106	5.9 (1.6;10.2)	(2.37;19.9)	0.016
**Employed**						
No	16/431	3.5 (0;7.2)	54/622	7.3 (4.3;10.3)	(-6.62;-0.89)	0.014
Yes	87/596	14.2 (9.9;18.5)	124/957	10.2 (7.5;12.9)	(0.48;7.56)	0.021
**Marital status**						
Never married	72/1191	5.7 (2.2;9.2)	122/1282	6.5 (3.3;9.8)	(-2.73;1.2)	0.478
Married/Living in union	27/99	29.1 (18.1;40.2)	20/147	16.8 (9.0;24.6)	(0.61;23.96)	0.034
Separated/divorced/Widowed	15/85	19 (9.5;28.5)	18/74	25.1 (14.1;36.1)	(-21.08;7.38)	0.396
**Religion**						
Christian	87/1018	8.3 (5.1;11.6)	128/1055	9.1 (5.6;12.6)	(-3.37;1.67)	0.544
Muslim	15/258	6.1 (1.5;10.7)	22/305	6.3 (2.9;9.7)	(-4.06;4)	0.876
Other/No religion	12/100	10.2 (1.9;18.5)	28/220	12.6 (7.5;17.7)	(-10.8;5.35)	0.608
**Sexual Identity**						
Bisexual	38/529	8.3 (4.4;12.1)	49/831	5.6 (3.75;7.4)	(0.68;14.41)	0.071
Gay/Homosexual	53/642	7.1 (3.7;10.5)	104/582	14.9 (11.1;18.8)	(-0.32;5.65)	< 0.001
Heterosexual	15/141	10.2 (3.5;16.8)	4/84	2.9 (0;6.2)	(-11.46;-4.1)	0.063
Other	3/23	6.8 (0;15.86)	19/80	15.6 (8.52;22.59)	(-25.81;8.2)	0.666
**City**						
Beira	53/581	9.5 (1.0;17.9)	42/526	8.1 (0;19.9)	(-2.23;4.81)	0.516
Maputo	50/447	9.3 (3.2;15.5)	104/526	14.7 (7.4;21.9)	(-9.5;-0.98)	0.017
Nampula	11/351	3.9 (0;14)	32/528	5.7 (3.1;8.3)	(-4.77;1.39)	0.332
**Age at first anal intercourse**						
< 15	15//114	11.8 (5.24;18.44)	28//108	14.2 (8.24;20.24)	(-12.14;7.17)	0.722
15–17	21//339	4.5 (1.3;7.71)	52//445	8.5 (5.23;11.71)	(-7.77;-0.46)	0.033
18–20	19//523	3.7 (0;7.5)	51//574	6.5 (3.43;9.48)	(-5.57;-0.06)	0.048
21+	52//344	16.2 (11.09;21.29)	37//304	15 (9.68;20.4)	(-4.77;7.07)	0.77
**Number of male sexual partners in the last 12 months prior to the survey**						
<=1	62/713	9.1 (5.8;12.3)	35/375	9.9 (6.2;13.6)	(-4.72;4.13)	0.769
2	22/384	5.2 (1.3;9.1)	47/277	14.4 (9.4;19.3)	(-9.31;-0.97)	< 0.001
3+	25/228	10.1 (5.4;14.7)	58/342	12.3 (8.4;16.3)	(-3.32;5.96)	0.501
**Use lubrication during anal sex**						
Never	53/792	6.7 (3.8;9.5)	17/364	5.1 (2.3;7.8)	(-1.6;4.54)	0.406
Sometimes	29/282	10 (5.1;15.0)	46/576	7.3 (4.8;9.8)	(-1.71;6.99)	0.233
Always	32/296	10.8 (5.4;16.2)	107/574	14 (10.4;17.6)	(-7.91;1.66)	0.231
**Gave money, service or good in exchange for sex in the last year**						
No	92/1130	8.0 (4.7;11.2)	140/1010	10.6 (8.0;13.2)	(-5.19;-0.07)	0.043
Yes	16/188	9.4 (3.3;15.5)	25/409	6.1 (3.3;8.8)	(-1.73;8.65)	0.177
**Received money, service or good in exchange for sex in the last year**						
No	60/817	6.7 (3.1;10.3)	86/615	10.4 (7.5;13.3)	(-6.78;-0.57)	0.017
Yes	48/501	10.5 (6.4;14.6)	79/805	8.5 (5.7;11.2)	(-1.34;5.6)	0.233
**AUDIT-C**						
Problematic	60/658	9.2 (5.61;12.73)	108/821	11 (8.21;13.78)	(-4.78;1.64)	0.098
Not- Problematic	53/664	7.5 (4.47;10.57)	70/759	7.9 (5.21;10.63)	(-2.73;3.03)	0.878
**Self-reported STI in the last year**						
No	80/1191	6.6 (3.1;10.1)	138/1304	8.4 (5.2;11.5)	(-3.95;0.34)	0.104
Yes	34/185	17.5 (10.7;24.2)	40/271	12.5 (7.5;17.5)	(-2.43;11.93)	0.245
**HIV tested in the last year**						
No	77/843	8.6 (5.6;11.5)	75/491	11.5 (8.15;14.7)	(-6.41;0.68)	0.106
Yes	37/533	7.1 (3.3;10.9)	90/782	9.9 (7.05;12.7)	(-5.9;0.46)	0.107
**Risk perception of HIV infection**						
No risk	23/400	5.3 (1.5;9.0)	8/166	3.7 (0.1;7.3)	(-2.37;5.64)	0.539
less risk	27/508	4.2 (0.4;7.9)	32/511	6.1 (3.2;8.8)	(-4.83;0.96)	0.208
Moderate	27/254	10.9 (5.3;16.5)	18/321	3.4 (1.5;5.4)	(2.91;12.28)	0.001
Elevated	23/154	15.9 (7.8;23.9)	19/177	10.4 (4.4;16.5)	(-2.45;13.28)	0.19
**Circumcised**						
No	65/493	11.7 (7.6;15.8)	37/139	25.1 (16.7;33.4)	(-21.63;-5.2)	< 0.001
Yes	49/883	5.9 (2.2;9.6)	141/1441	7.8 (3.6;11.9)	(-4.05;0.29)	0.102
**Sought health care in the last year**						
No	41/697	5.4 (2.31;8.49)	78/872	7.2 (4.8;9.6)	(-4.31;0.76)	0.187
Yes	73/679	10.7 (7.2;14.14)	100/705	11.3 (8.1;14.5)	(-4.04;2.85)	0.789

### Observed changes in HIV prevalence among MSM by city

Table 2 illustrates alterations in the odds of HIV infection between 2011 and 2020 for three cities. For Maputo city, the results shows that the odds of HIV infection were about two times higher in 2020 than in 2011. While in Beira and Nampula city, results show that there was not a significant change in the risk of HIV infection between 2011 and 2020. The results indicate a notable overall rise in HIV prevalence between 2011 and 2020, where MSM in 2020 were 1.4 times more likely to be infected by HIV when compared to MSM in 2011 (Table 2).

**Table 2:** Logistic regression showing the change in the odds of HIV infection between 2011 and 2020

City	Crude Odd Ratio (95% CI)	*p*-value
Maputo	2.0 (1.4–2.8)	< 0.001
Beira	0.9 (0.6–1.3)	0.500
Nampula	2.0 (1.0–4.0)	0.053
**Overall**	**1.4 (1.1–1.8)**	**0.007**

### Multivariate analysis

The results for the multivariate logistic regression are presented in Table 3. This table reveals a notable interaction between the “year” variable and specific risk factors, highlighting the changing dynamics in HIV risk among MSM in relation to these factors over a nine-year timeframe.

The findings reveal that after accounting for other covariate, the odds of HIV infection remained constant over the nine-year within each site. Additionally, the results reveal that the likelihood of HIV infection in Beira City is 0.4 times lower than in Maputo City. Furthermore, the results show that in 2011 BBS, MSM aged 25 years old or above were about 13 times more likely to be HIV positive than those aged between 18 and 24 years old, whereas in 2020 BSS this reduced by 80% to a factor of 2.8. The results also show that married MSM are 2.4 times more likely to infected by HIV than MSM who never married. Moreover, the likelihood of HIV infection remained consistent across the surveyed periods. With respect to age at first anal intercourse, the results reveal that risk of HIV infection decreases as the age at first anal intercourse increases, and this pattern is consistent across the surveys.

The results further show that in 2011 BBS, MSM who reported having two male sex partners in the last 12 months were five times less likely to be HIV positive than those who reported have one partner. In 2020 BBS, it is observed that the odds increased by 72% to a factor of 0.72, indicating that in 2020 BBS MSM who reported having two male partners were 28% less likely to be HIV positive than those who reported having one male partner. We further observe that in 2011 BBS, MSM who gave money and services in exchange of sex were 1.3 times more likely to be HIV positive, whereas in 2020 BBS MSM who reported having given money, goods, or services in exchange for money were found to be 63% less likely to be HIV positive.

The findings also show that MSM who have ever had a STI diagnosis or symptoms are more likely (OR = 1.9, 95% CI: 1.6; 2.3) to be infected with HIV, which is consistent across both surveys. Furthermore, MSM who have ever sought the advice of a health expert are 1.7 times more likely to be infected with HIV than those who have never sought the advice of a health professional. The results also show that the odds of being infected with HIV is 60% lower for circumcised MSM than uncircumcised MSM. This underscores a substantial protective effect of circumcision against HIV transmission, manifesting in a markedly lowered risk observed across both survey years.

The current analysis further indicates that MSM who tested for HIV and received the test results 12 months before the survey were two times less likely (c) to be HIV positive than those who did not test for HIV 12 months before the survey. The analysis further indicates that circumcised MSM have a 60% lower likelihood of being HIV positive compared to uncircumcised MSM, highlighting a significant protective effect of circumcision against HIV transmission. Finally, the results suggest that MSM who consistently use lubricants for anal sex have approximately three times higher odds of being HIV positive.

**Table 3:** Multivariate logistic regression parameter estimates. The estimates are presented in the odds ratio (OR) scale accompanied by 95% confidence interval in brackets

Variables	OR (95% CI)	*P*-value
**(Intercept)**	0.1 (0.0-0.3)	< 0.001
**Age (ref = 18–24)**		
25+	13.8 (11.4–16.6)	< 0.001
**Year (ref = 2011)**		
2020	2.6 (1.2–5.6)	0.015
**Marital Status (ref = Never married)**		
Married/living in union	2.4 (1.4–4.1)	< 0.001
Separated/divorced/widowed	1.6 (0.9–2.9)	0.106
**Worked in the last 12 months prior to the survey (ref = No)**		
**Yes**	1.9 (1.6–2.4)	< 0.001
**Sexual identity (ref = Heterosexual)**		
Bisexual	0.6 (0.3–1.2)	0.147
Gay/homosexual	2 (0.9–4.2)	0.053
Other	2 (0.7–5.2)	0.147
**Province (ref = Maputo)**		
**Beira**	0.4 (0.3–0.6)	< 0.001
**Nampula**	0.8 (0.4–1.3)	0.327
**Age at first anal intercourse (ref = < 15)**		
15–17	0.5 (0.3–0.8)	0.007
18–20	0.3 (0.2–0.5)	< 0.001
21+	0.2 (0.1–0.4)	< 0.001
**Number of male sexual partners in the last 12 months prior to the survey (ref= <=1)**		
2	0.4 (0.2–0.8)	0.009
3+	0.6 (0.3–1.2)	0.129
**Received money and services in exchange for sex in the last 12 months (ref = No)**		
Yes	1.3 (0.6–2.6)	0.542
**Ever had a diagnosis or symptoms of STI in the last 12 months prior to the survey (ref = No)**		
Yes	1.9 (1.6–2.3)	< 0.001
**Ever looked for health professional (ref = No)**		
Yes	1.7 (1.2–2.4)	0.002
**Tested and received results in the 12 months prior to the survey (ref = No)**		
Yes	0.5 (0.4–0.7)	< 0.001
**Circumcision (ref = No)**		
Yes	0.4 (0.3–0.6)	< 0.001
**How often do use lubrication for anal sex (ref = Never)**		
**Sometimes**	1.5 (0.9–2.4)	0.082
**Always**	2.8 (1.7–4.4)	< 0.001
Age x Year		
25 + X Year	0.2 (0.1–0.4)	< 0.001
**Number of male sexual partners in the last 12 months prior to the survey (ref=<=1) X Year**		
2 X Year	3.7 (1.5–9.3)	0.006
3 + X Year	2 (0.8–4.8)	0.125
**Gave money, goods, services in exchange for sex X Year**	0.3 (0.1–0.7)	0.006

## Discussion

Worldwide MSMs are known to act as an important key population in the global dynamics of HIV sexual transmission [4]. As far as we know, this is the first study conducted in Mozambique to analyses the trend HIV prevalence among MSM. Our findings indicate an increase in HIV infection rates Maputo City, observed between the first round of BBS conducted in 2011 and the subsequent round conducted in 2020, while there was no significant change in Beira City and Nampula. The observed HIV prevalence trend is consistent with the prevalence trend in general population of adults. In fact, data from the last two AIDS Indicator Surveys (IMASIDA 2015 and INSIDA 2020) revealed that the prevalence of HIV among men aged 15–19 years old in Maputo City has increased from 12.3% (2009) to 22.3% (2015) [13]. In Sofala province, where Beira city is located, the HIV prevalence has varied from 12.6 to 16.3%, and in Nampula the HIV prevalence has shift from 3.3% in 2009 to 5.7% in 2020 [5].

The HIV burden is more pronounced among older MSM in contrast to their younger counterparts. Eluwa et al., in their study on the increased HIV prevalence among MSM in Nigeria reported a similar trend of our findings [14]. In a previous study conducted in Mozambique young key populations are more aware of their HIV risk compared to their counterparts in the general population [15]. In 2016 the Mozambique Ministry of Health implemented *National Guidelines for the Integration of Prevention, Care and Treatment Services for Key Populations*. These guidelines established a comprehensive package of services for the key population, resulting in significant achievements in several key indicators. In addition, the guidelines also facilitated the provision of high-quality, user-friendly, integrated, and comprehensive community and facility-based interventions across the HIV Continuum of Prevention to Care and Treatment. As a result, younger key populations individuals are being diagnosed with HIV and successfully linked to HIV medical care.

Our findings suggest that sexual identity did not lead to the difference in risk for seropositivity in the last 9 years (2011 to 2020). However, in the 2020 BBS, bisexual individuals exhibited a reduced likelihood of HIV infection. This observation raises the hypothesis that individuals who identify with a sexual orientation involving partners from both gender groups might be more inclined to engage in occasional relationships [16]. A previous study conducted in the same country revealed that bisexual MSM might experience social isolation from other MSM. Due to the lack of pertinent information and support, this group could unintentionally contribute to the heightened long-term risk of HIV transmission [17].

Drawing from the outcomes of our analysis, we have identified that the practice of exchanging sex for monetary compensation or other material goods holds a noteworthy association with HIV infection. This connection underscores the economic vulnerability that is prevalent among individuals in low-income countries. This vulnerability can drive individuals to engage in transactional sex as a means to secure improved living conditions and address fundamental life necessities [3, 4], however our results did not find a statistically significant difference between the two BBS in the MSM population.

The correlation identified in our research between the count of sexual partners within the six months prior to the interview and the occurrence of HIV infection aligns with the outcomes reported in contemporary literature, including similar results in other studies using RDS and showed a significantly increasing trend. It is widely acknowledged that a substantial number of sexual partners serves as a risk factor for engaging in behaviors that heighten the vulnerability to HIV infection within the MSM population. This substantiates the rationale for the heightened probability of HIV infection within this demographic. Consequently, there exists a compelling necessity to enhance interventions aimed at mitigating risk behaviors among MSM.

Our analysis of trends in HIV risk behaviors showed an increased prevalence of HIV among uncircumcised MSM between the two surveys. This trend underscores the ongoing relevance of circumcision as an effective measure for reducing HIV risk, even within the context of same-sex relationships [18, 19]. As we strive to address the evolving landscape of HIV transmission, these insights highlight the necessity of promoting accurate information about circumcision, safe sexual practices, and equitable access to sexual health resources for MSM.

One of the strengths of this study is that it represents the first comprehensive analysis of HIV prevalence trends among MSM in Mozambique. By conducting surveys in multiple cities and across different time points, we were able to capture variations in HIV prevalence that may not have been evident in smaller, localized studies. This broad geographic scope enhances the generalizability of our findings to the larger MSM population in Mozambique.

Additionally, our study employed the RDS method, which is designed to overcome some of the challenges of sampling hidden populations like MSM. RDS allows for the estimation of population-level statistics, making our results more robust and informative. Second, it’s worth noting that there is no definitive ‘gold standard’ for directly comparing the outcomes of such studies. The majority of global data on MSM originates from convenience samples or the random selection of specific locations and timeframes where MSM commonly interact. While our study contributes valuable insights, it underscores the need for a comprehensive expansion of healthcare services and robust HIV surveillance tailored to key populations. The emergence of the COVID-19 pandemic posed significant challenges during the implementation of the second round of the 2020–2021 survey. Delays and mobility constraints in some cities were influenced by the pandemic’s emergency context. Social distancing protocols, implemented to contain the spread of COVID-19, may have discouraged some individuals from participating in the survey.

## Conclusion

In conclusion, our findings demonstrate a significant increase in HIV prevalence among MSM in Maputo City and Nampula from 2011 to 2020, underscoring the urgency for targeted interventions to address the unique needs and complexities faced by this demographic. This prevalence trend persists among MSM aged 25 years and older. The results also underscore the importance of socioeconomic factors, such as engagement in transactional sex, in driving HIV transmission among MSM. Implementing comprehensive and culturally sensitive approaches that tackle stigma, discrimination, and economic vulnerabilities is crucial for reducing HIV risk and promoting the overall health and well-being of MSM in Mozambique.

Furthermore, this study emphasizes the significance of a comprehensive, evidence-based approach to prevent HIV transmission among MSM. The trends identified in our study reveal an elevated risk of HIV infection among MSM, underscoring the necessity for further research to guide HIV prevention programs tailored to the social and cultural context, age, and religious beliefs of MSM in Mozambique. By strengthening the public health response to prevent and treat HIV infection among MSM, the objective of reducing the HIV infection rate within this population in Mozambique is attainable. Achieving this goal requires a concerted effort to engage with MSM communities, offering services and information that cater to their specific needs and addressing the various vulnerabilities they may face. Only through such targeted and empathetic approaches can we truly address the complexities of HIV prevention and care among MSM, ultimately fostering a healthier and more equitable society.

## Data Availability

All study information and datasets are fully accessible at the Mozambique National Institute of Health (INS) data repository for researchers who meet the criteria for accessing confidential data. The data originate from the BBS studies, and the authors can be contacted through: https://ins.gov.mz/institucional/unidade-organicas/direccoes/direccao-de-inqueritos-e-observacao-de-saude/bases-de-dados/.
